# Sex dependent imprinting effects on complex traits in mice

**DOI:** 10.1186/1471-2148-8-303

**Published:** 2008-10-31

**Authors:** Reinmar Hager, James M Cheverud, Larry J Leamy, Jason B Wolf

**Affiliations:** 1 Faculty of Life Sciences, University of Manchester, Manchester M13 9PT, UK; 2 Department of Anatomy and Neurobiology, Washington University School of Medicine, St Louis, MO 63110, USA; 3 Department of Biology, University of North Carolina at Charlotte, Charlotte, North Carolina 28223, USA

## Abstract

**Background:**

Genomic imprinting is an epigenetic source of variation in quantitative traits that results from monoallelic gene expression, where commonly either only the paternally- or the maternally-derived allele is expressed. Imprinting has been shown to affect a diversity of complex traits in a variety of species. For several such quantitative traits sex-dependent genetic effects have been discovered, but whether imprinting effects also show such sex-dependence has yet to be explored. Moreover, theoretical work on the evolution of sex-dependent genomic imprinting effects makes specific predictions about the phenotypic patterns of such effects, which, however, have not been assessed empirically to date.

**Results:**

Using a genome-scan for loci affecting a set of complex growth and body composition traits from an intercross between two divergent mouse strains, we investigated possible sex-dependent imprinting effects. Our results demonstrate for the first time the existence of genomic imprinting effects that depend on sex and are not related to sex-chromosome effects. We detected a total of 13 loci on 11 chromosomes that showed significant differences between the sexes in imprinting effects. Most loci showed imprinting effects in only one sex, with eight imprinted effects found in males and six in females. One locus showed sex-dependent imprinting effects in both sexes for different traits. The absence of an imprinting effect in one sex was not necessarily indicative of the overall inactivity of the locus in that sex, as for several loci a significant additive or dominance effect was detected. Moreover, three loci exhibited significant additive effects in both sexes but their imprinting effect was restricted to one sex.

**Conclusion:**

Our results clearly show that imprinting effects can be sex-dependent and also suggest that new candidate imprinted loci can be detected when taking account of sex-specific imprinting effects. However, predictions made about the evolution of sex-dependent imprinting effects and associated phenotypic patterns cannot be unequivocally supported at present and further research into the selection pressures applied to the strains of mice used in our study is required.

## Background

While most genes are expressed from both the paternally and maternally derived allele, imprinted loci show parent-of-origin-dependent gene expression such that either the paternal or the maternal allele only is expressed [1,2]. This monoallelic pattern of gene expression is usually caused by differential methylation of the paternal and maternal allele [3] and results in phenotypic differences between the two heterozygotes (where the only difference is the parent-of-origin of the parental alleles) at a given locus [4]. Since the discovery of genomic imprinting over twenty years ago [5], to date over 80 imprinted genes and their associated phenotypic effects have been identified and described in mice (http://www.har.mrc.ac.uk/research/genomic_imprinting; October 2008). Many imprinted genes have been shown to directly or indirectly affect resource transfer between mother and offspring [6-8] and thus individual development and growth. The traits affected by genomic imprinting are widespread and are found predominantly in mammalian systems, suggesting that imprinting has evolved for different reasons at different loci, or may be maintained by selection because it serves different functions at different stages in development and in different tissues [2,9]. Among traits affected by imprinting are weight-related [8] and body composition traits [10] cognitive abilities [11,12] and associated disorders (e.g. Prader-Willi and Angelman syndrome), and obesity [7,13-16], highlighting the impact of epigenetic sources to variation in complex traits.

### Sex-dependent genetic effects

Previous research has demonstrated that patterns of genetic variation such as additive and dominance effects (the former being a measure of the independent effects of alleles, generally defined as half the difference between the average phenotypes of homozygotes, and the latter being a measure of interactions between alleles at a locus, and defined as the deviation of the average phenotype of the heterozygote from the midpoint between that of the two homozygotes) of loci can show considerable differences depending on the sex of the individual [17,18]. Such sex-dependent genetic effects have been reported for a number of different traits, including blood pressure [19], longevity in humans [20] and *Drosophila *[21], bodyweight [22,23], and morphology in mice [24], and various disease-related traits in humans [25,26]. However, we are not aware of any study that has explored whether autosomal sex-specific genomic imprinting effects exist, although sex-dependent imprinting effects have been suggested for the X chromosome [27,28] as a potential explanation for disorders such as Turner syndrome and autism [29-31]. Thus, we currently know very little about the likelihood and patterns of differences in imprinting effects in males and females.

### Evolutionary and genetic significance of sex-dependent imprinting effects

Sex-dependent imprinting effects are of interest both from a genetic and an evolutionary perspective. For example, analyses of sex-dependent imprinting effects might reveal loci that either show imprinting in only one sex but bi-parental expression of alleles in the other sex. Alternatively, loci could be imprinted in both sexes but show different patterns of imprinting (e.g., maternal expression in one sex but paternal expression in the other). Such loci would suggest flexibility in expression patterns and possibly the underlying imprinting mechanism that may not be expected from current models of imprinting. It is also possible that some loci might show sex-dependent imprinting because they exhibit strictly sex-limited expression, where expression is limited to either males or females. Given that the sexes across taxa often differ considerably but are virtually genetically identical except for the sex chromosomes, sex-dependent gene expression has been predicted to be a relatively common phenomenon and its existence has been demonstrated in a number of different systems [18,32]. Therefore, we may expect *a priori *that some imprinted loci would show sex-dependent expression.

From an evolutionary perspective, sex-dependent imprinting effects are of particular interest because theory predicts the existence of such differences in imprinting between the two sexes [33] when selection acts differentially on traits expressed in males versus females (e.g. on loci affecting size dimorphism). Specifically, Day and Bonduriansky [33] predict silencing of the allele derived from the sex that does not experience selection. For example if males are under selection for increased body size but females are not, loci affecting body size are predicted to show paternal expression in males. Moreover, sex-dependent imprinting effects might act in same manner as has been suggested for sex-dependent genetic effects [[34] but see [35]] to help maintain genetic variation because selection may favour alternative alleles or imprinting patterns in males and females.

### Aims

In this study, we set out to explore whether sex-dependent imprinting effects on complex traits exist in mice by investigating sex differences in the imprinting effects of quantitative trait loci (QTL) in males and females. We characterize the phenotypic patterns caused by genomic imprinting in males and females separately and also determine whether such loci exhibit sex-specific additive or dominance effects. Furthermore, we estimate the proportion of phenotypic variance explained by sex-dependent imprinting effects. Results demonstrate that genomic imprinting effects can be different between the sexes and reveal the existence of 13 QTL with such patterns located on 11 autosomal chromosomes.

## Results

Overall, we detected a total of 13 QTL on 11 chromosomes affecting both body and organ weight measures that showed a significant interaction of sex and genomic imprinting effect (*Sbi*QTL, where '*Sbi*' refers to *s*ex *b*y *i*mprinting interaction). For all QTL, we confirmed that these sex interactions were true genomic imprinting effects rather than maternal genetic effects (see Methods).

Nine of the ten measured weekly bodyweights were affected by an interaction between sex and imprinting as was postweaning growth (Additional file 1). In addition, the QTL exhibiting sex-dependent imprinting effects also significantly affected reproductive fatpad, spleen, kidney, heart and liver weights (Additional file 1). Two loci (*Sbi15.1 *and *Sbi17.1*) were found to affect all weight traits except for week 1 and week 3 weight, whereas six other *Sbi*QTL affected between one and seven weight traits each. Four *Sbi*QTL influencing organ weights, by contrast, showed an effect on only one specific trait. Focusing on the traits themselves, we found that week 5 body weight was modulated by the highest number of *Sbi*QTL (seven) followed by week 4 weight (six). Organ weights were generally affected by fewer *Sbi*QTL. While liver weight was affected by two *Sbi*QTL, all other organ traits were found to be influenced by three loci.

### Sex-dependent genomic imprinting effects

**Figure 1:**
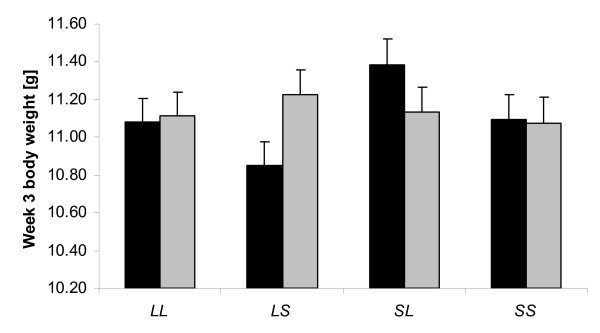
**Genomic imprinting effects at *Sbi3.1 *for week 3 body weight in males (black bars) and females (grey bars) for the four ordered genotypes.** There is no difference between female heterozygotes but a significant difference in males showing a bipolar imprinting pattern. Shown are the mean trait values for the four ordered genotypes with their standard errors (SE).

**Figure 2:**
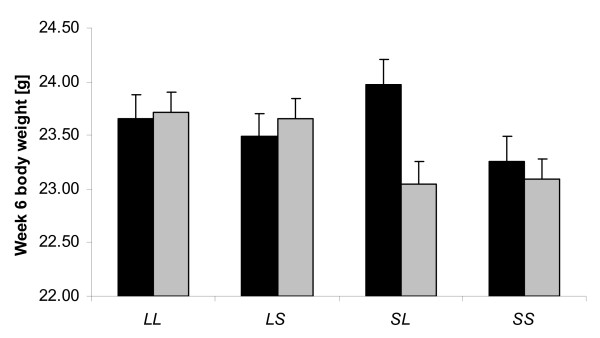
**Genomic imprinting effects at *Sbi6.1 *for week 6 body weight in males (black bars) and females (grey bars) for the four ordered genotypes. **Here, there is no difference between male heterozygotes but a significant difference in females showing a paternal imprinting pattern. Shown are the mean trait values for the four ordered genotypes with their standard errors (SE).

For most loci, the interaction effect was due to the imprinting effect occurring in one sex only. At three loci, the imprinting effect was restricted to females for all affected traits and in five other loci this effect was found in males only. For example, *Sbi3.1 *showed a bipolar imprinting pattern in males but has no imprinting effect in females (Figure 1) whereas at *Sbi6.1 *no imprinting effect occurred in males but only in females with a paternal expression pattern (Figure 2). At several loci, no significant imprinting effect in either sex was found for some or all traits despite the fact that the locus shows a significant interaction effect (i.e., a significant difference between the sexes in the imprinting effect; see Additional file 2). In these cases, the estimated imprinting effects are opposite in sign to each other in the two sexes but not strong enough in either sex to be significant. *Sbi15.1 *showed a somewhat different pattern where the imprinting effect occurred in both sexes for separate traits. While the sex by imprinting interaction effect was very strong for many traits for this locus (exceeding genome-wide significance levels), the imprinting effects in the separate sexes were often only marginally significant.

Interestingly, the fact that an imprinting effect occured in one sex only is not necessarily due to the overall inactivity of the locus in the other sex (i.e. no detectable additive or dominance effect). Rather, at four loci that do not show an imprinting effect in males, we found a significant additive or dominance effect in males (Additional file 2). The reverse was found in females only at two loci. Another indication that overall sex-specific expression of the locus does not sufficiently explain the occurrence of sex-dependent imprinting effects is given by the fact that three loci displayed a significant main additive effect in *both *sexes but the imprinting effect was restricted to either males or females.

### Imprinting patterns and variance explained

The imprinting patterns found at a locus can generally be classified into three canonical categories (though other more complex patterns that deviate from these canonical patterns can occur), parental expression (paternal or maternal expression), bipolar dominance and polar over- or underdominance [10,36], depending on the pattern of variation among the four ordered genotypes at a locus (i.e., alleles are ordered by the parent-of-origin of alleles, thus allowing the distinction between the alternative heterozygotes). Parental expression occurs when the genotypes sharing the same allele inherited from the same parent have the same phenotype. For example, with paternal expression, individuals that inherited the same paternal allele show the same average phenotype. Bipolar dominance is defined as the pattern when the two heterozygotes are significantly different from each other but the two homozygotes are not, such that the genotypic value of one heterozygote is larger than that of the homozygotes while the other is smaller. Finally, polar over- or underdominance is defined by the pattern where one of the two heterozygotes is significantly different from the three other genotypes (either larger, in the case of polar overdominance or smaller, in the case of polar underdominance), which are not significantly different from each other. Where imprinting appeared in only one sex, we found paternal and maternal expression, polar under- and overdominance and bipolar expression (Additional file 1). Among the loci that showed imprinting in males only, four showed paternal expression, three bipolar dominance and one polar overdominance where the *SL *heterozygote was larger than all other three genotypes. Imprinting patterns in females were paternal expression for three loci, and polar over- and underdominance as well as bipolar dominance once each in females.

The proportion of phenotypic variance explained by the imprinting and sex interaction ranged from 0.58% to 3.6% depending on the trait (Additional file 1). For example, the interaction at *Sbi6.1 *accounted for 3.28% of phenotypic variance of week 4 body weight but this value decreased to just 0.75% for week 10 body weight.

## Discussion

The most important results emerging from our study are firstly that genomic imprinting effects on complex traits can depend on sex and, secondly, that new loci can be found in sex-specific analyses that may escape detection in searches for imprinting effects in the population as a whole. Our analysis of sex-dependent imprinting effects on complex traits uncovered a total of 13 *Sbi*QTL where often the imprinting effect was restricted to one sex only. Comparing the locations of these *Sbi*QTL with previously-detected imprinted QTL, i.e. those that exert a significant main effect on a given trait [10,36], many QTL found here are novel loci with the exception of *Sbi7.1 *and those located on the proximal region on chromosome 3, which are within the confidence regions of previously described loci. This is a particularly important result since it suggests the existence of more imprinted loci that would remain undetected if males and females were not differentiated.

### Imprinting patterns

Our study also showed interesting imprinting patterns in the separate sexes. At five loci, imprinting effects were detected in males only while at three loci such effects were found in females only. In the vast majority of cases, the sign of the imprinting effect was opposite in the two sexes at loci in which the imprinting effect occurred in one sex only (e.g. *Sbi1.1*: negative in males and positive in females, see Additional file 2). Overall, the imprinting effect patterns for a given sex were diverse, ranging from parental (paternal or maternal) expression to bipolar dominance and polar over- or underdominance. We note that when an imprinting effect was limited to one sex only, other genetic effects (additive and dominance) may still have an effect in the other sex or in both sexes. This suggests that the overall expression of the locus is not sex-specific but rather that the imprinting effect alone is sex-dependent.

### Comparison with genetic effects

Compared to the occurrence of sex-dependent additive and dominance effects reported in the F_2 _SM/J × LG/J cross [23], sex-dependent imprinting effects were more common for weight and growth traits but each individual locus was found to affect fewer traits. Vaughn et al. [23] report five QTL on chromosomes 9, 12, 15 and 16 that showed significant sex-specific additive and dominance interactions for 11 different weight and growth traits with a total of 27 traits affected by all loci (one trait can be affected by multiple loci). This compares with eight *Sbi*QTL affecting a total of nine weight and growth traits where seven loci affected two or more traits each. Of the five loci with sex-dependent additive or dominance effects found by Vaughn et al. [23], four had an additive and dominance effect in males and were not expressed in females at all (i.e. they showed neither a significant additive nor a dominance effect) whereas on chromosome 16 there was a male-specific QTL at the proximal part of the chromosome and a female-specific QTL on the distal portion of the chromosome. Vaughn et al.'s [23] findings differ from ours in that a locus that does not show an imprinting effect in one sex can still have additive effects in the same sex or even both sexes. This latter result may be somewhat surprising, because it suggests that such loci do not show simple sex-specific expression in the sense that overall expression is restricted to one sex. Rather, it appears that the imprinting mechanism is not acting in one sex. Perhaps differential methylation at the locus in males and females accounts for this observation, or there may be some *trans *control of expression that depends on sex.

### Genome locations of SbiQTL

With the exception of *Sbi6.1 *the locations of our detected *Sbi*QTL do not map to regions where currently known imprinted genes are located http://www.geneimprint.com, although we note that the confidence intervals of *Sbi15.1 *and *Sbi7.1 *are just outside of a large imprinting cluster on chromosome 7 and the region containing *Peg13 *on chromosome 15 (Additional file 2). The confidence region for *Sbi6.1 *on chromosome 6 is close to a cluster with several imprinted genes, amongst them paternally expressed gene 10 (*Peg10*) and *Sgce *[37]. In the light of our finding that imprinting effects can be sex-dependent it might be worthwhile to investigate whether known imprinted genes such as the aforementioned show similar effects, perhaps by comparing male and female phenotypes of knock-out mutants separately or by looking for differences in expression levels.

It should be noted that the primary objective of this study was not to find imprinted loci *per se *(see [10,36] for details on these analyses) but rather to search specifically for sex by imprinting interaction effects. In a simulation study, Luedi et al. [38] predicted a total of 600 imprinted genes across the genome. While some congruence of our confidence intervals and the locations of predicted genes might be expected by chance, the loci identified in this study may yield novel candidate gene locations that could be identified in fine mapping or gene expression studies.

### The evolution of sex-dependent imprinting effects

One possible mechanism underlying our findings involves the role of modifier loci that regulate the expression of imprinted loci affecting the measured traits. In this scenario, the expression of the modifier locus is sex-specific (e.g. comparable to sex-specific effects of additive loci as reported e.g. in Vaughn et al. [23] and occurs during gametogenesis in *offspring*. Day & Bonduriansky [33] invoke such 'sexually dimorphic imprinting' as part of an intralocus sexual conflict model to explain the evolution of genomic imprinting. Assuming that some loci have positive fitness effects when expressed in one sex but negative when expressed in the other, imprinting is predicted to evolve because selection favours silencing of loci in the sex in which these loci are not under directional selection. Among the traits with potential sexually antagonistic effects proposed by Day & Bonduriansky [33] is growth because size dimorphism observed in many species suggests that loci modulating growth may be under sex-specific selection. Applied to loci that affect growth and assuming antagonistic selection working on males and females, one would predict from this hypothesis that such loci should show paternal expression in males and maternal expression in females. While mice in general show sexual size dimorphism and are, under natural conditions, likely to be under selection for larger males to some degree, the specific selective regime applied to the strains used in our study does not suggest a sex difference. Our results do not show that maternal expression is more common in females than in males and paternal expression is found equally often in males and females and the pattern seen at loci affecting growth (*Sbi3.2 *and *Sbi4.1*) is bipolar dominance in males. Thus, at present it seems rather more difficult to assess the predictions made using our data, in particular in the absence of clear evidence for differential selection in males and females in the founding lines.

## Conclusion

Results of our study showing sex-dependent imprinting have not been previously reported to our knowledge. Nonetheless, existing data could be analysed to investigate whether such effects occur in other populations. For example, at the phenotypic level one could compare the growth of male and female individuals carrying a mutation at an imprinted locus (e.g. [8]) to the growth of the wildtype and explore whether the growth difference is proportionally greater in one sex than the other. It may also be possible to directly compare differences in expression levels of imprinted genes in both sexes in a given tissue.

We furthermore demonstrate that candidate imprinted loci can be found by analysing sex-specific imprinting effects as opposed to pooled data because the sign of effect can be opposite in the two sexes and phenotypic patterns can be different. The latter also has important implications for research on disease-related phenotypes associated with dysfunctional parent-of-origin-dependent effects and future research in this area should consider sex-dependent imprinting effects.

Further theoretical studies are required to investigate whether sex-specific imprinting effects can be predicted as a result of asymmetrical fitness effects on patrilineal versus matrilineal relatives as outlined by the currently favoured hypothesis for the evolution of imprinting, the conflict or kinship theory of genomic imprinting [39,40]. In addition, our results should encourage future theoretical work to address the unresolved issue of whether sex-dependent imprinting effects might play a role in the maintenance of genetic variation.

## Methods

### Study population and phenotypes

In this study we analysed variation in the F_3 _generation of an intercross between the Large (LG/J) and Small (SM/J) inbred mouse strains [41] that were selected for either large or small body weight at 60 days of age [42]. These strains differ by 6–8 standard deviations in size and growth related traits [41] and therefore represent an excellent model system to study imprinting effects arising from genes regulating growth and development. The study population was generated by first mating ten males of the SM/J strain to ten females of the LG/J strain resulting in the F_1 _population of 52 individuals. These F_1 _individuals were randomly mated to produce 510 F_2 _animals, representing our parental generation. Random mating among F_2 _animals yielded 200 full-sib families of the F_3 _generation with a total of 1632 individuals. Further details of the husbandry are given in Vaughn et al. [23].

The traits analysed in this study are weekly body weights taken in mice 1 to 10 weeks of age. These measures were used to calculate two growth periods: pre-weaning (weeks 1–3) and post-weaning growth (weeks 3–10). Growth was calculated as the difference between weekly weights (i.e., gain in body weight) such that, for example, the growth from week 1 to week 3 is the difference between week 3 weight and week 1 weight. Pups were weighed weekly using a digital scale with an accuracy of 0.1 g. In addition, we analysed heart, kidney, liver, reproductive fatpad and spleen weights. To obtain these organ weights, mice were sacrificed after 70 days of age or after having produced and reared their offspring to weaning (at three weeks of age). At necropsy, all mice were first weighed to obtain an overall measure of body size and then dissected and their organs weighed to the nearest 0.01 gram using digital scales. The effects of age at necropsy, sex, and litter size at birth were removed from the data and the residuals used in the analysis prior to gene mapping [41].

### Genotyping

DNA was extracted from livers of both the F_2 _and F_3 _individuals using Qiagen DNeasy tissue kits and samples were scored for 384 SNP markers using the Golden Gate Assay by Illumina, San Diego, USA. These markers were previously found to be polymorphic between the two strains http://www.well.ox.ac.uk/mouse/INBREDS/. Fifteen loci had to be excluded from the analysis because they were not reliably scored. In addition, 16 loci were scored on the X chromosome, but were not included in this analysis because the statistical model for the X chromosome is currently unresolved. Thus, in total we analysed 353 loci across the 19 autosomes in this study. A genetic map of these markers in cM was produced using R/QTL and validated against the genome coordinate locations in the Ensembl database http://www.ensembl.org. The average map distance between markers in the F_2 _generation was 4 cM. Markers were reasonably evenly located throughout the genome except for several regions in which LG/J and SM/J strains have been found to be monomorphic [43].

### Haplotype recontruction

Both parental and offspring genotypes were used to reconstruct haplotypes for all mice using PedPhase [44], which produced a set of unordered haplotypes for the F_2 _parents and a set of ordered haplotypes (i.e. ordered by parent-of-origin of alleles) for their F_3 _offspring. Thus, we were able to distinguish the four possible genotypes at a given locus, *LL, SL, LS *or *SS *where the first allele refers to the paternally-derived allele and the second to the maternally-derived copy.

### Analysis of parent-of-origin-dependent effects

The four ordered genotypes at the marker loci (*LL, LS, SL *and *SS*) were assigned additive (*a*), dominance (*d*) and parent-of-origin (*i*) genotypic index scores following Mantey et al. [45]. In matrix form, these index scores are given by:

$$\left[\begin{array}{c} \bar{LL}\\ \bar{LS}\\ \bar{SL}\\ \bar{SS} \end{array}\right]=\left[\begin{array}{cccc} 1 & 1 & 0 & 0\\ 1 & 0 & 1 & 1\\ 1 & 0 & 1 & -1\\ 1 & -1 & 0 & 0 \end{array}\right]\left[\begin{array}{c} r\\ a\\ d\\ i \end{array}\right]\text{yielding estimates of the parameters}:\left[\begin{array}{c} r\\ a\\ d\\ i \end{array}\right]=\left[\begin{array}{c} \frac{\bar{LL}}{2}+\frac{\bar{SS}}{2}\\ \frac{\bar{LL}}{2}-\frac{\bar{SS}}{2}\\ \frac{\bar{LS}}{2}+\frac{\bar{SL}}{2}-\frac{\bar{LL}}{2}-\frac{\bar{SS}}{2}\\ \frac{\bar{LS}}{2}-\frac{\bar{SL}}{2} \end{array}\right]$$

The vectors of genotypic means are $\bar{LL}$, $\bar{LS}$, $\bar{SL}$, $\bar{SS}$,, *r *is the reference point for the model (the mid-point between homozygotes), *a *is the additive genotypic value (half the difference between homozygotes), *d *is the dominance genotypic value (the difference between the mean of the heterozygotes and the mid-point of the homozygote means), and *i *is the parent-of-origin or imprinting genotypic value (half the difference between heterozygotes) (cf. [45]).

These index scores were used to build a model for a genome scan for loci showing significant sex by parent-of-origin-dependent interaction effects (i.e. sex by *i *effects) using the Proc mixed Procedure in SAS 9.1; SAS Institute, Cary, NC, USA using maximum likelihood (see [46] for details). In this model, growth traits or organ weights are the dependent variables and sex, the additive, dominance and imprinting index scores as well as their interactions with sex were the fixed effects and family was a random effect. The mixed model with the fixed genetic effects and random family effect was used to scan the genome to produce a probability distribution for the overall effect of the locus as well as the sex by imprinting interaction effect. The probability was generated by comparing the -2 res log likelihood computed by SAS for the model with the fixed genetic effects and their interaction with sex with a reduced model that did not include these six genetic effects. The difference in the -2 res log likelihoods of the two models is chi-square distributed with 6 degrees of freedom (representing the fact that the two models differ by 6 fixed effects). These probability values were then transformed to a log probability ratio (LPR) in order to make them comparable to the LOD scores commonly seen in QTL analyses (LPR = -log_10 _[probability]).

Parent-of-origin effects can also appear as a result of maternal genetic effects rather than genomic imprinting [47]. Maternal genetic effects occur if the mother's genotype affects the offspring's phenotype beyond the effects of her genetic contribution to offspring phenotypes. Maternal effects can result in the appearance of parent-of-origin dependent effects because homozygous mothers can produce only one type of heterozygous offspring. By analogy, sex-dependent maternal effects can result in the appearance of sex by *i *effects. Therefore, we tested all loci with a significant sex by *i *interaction effect to confirm that the effect was not caused by a sex-dependent maternal genetic effect [cf. [47]]. Using our mixed model framework, we have advanced on our previous approach by testing whether the sex by *i *effect is dependent on whether individuals are reared by homozygous mothers (where a maternal effect could result in the appearance of a parent-of-origin dependent effect) or heterozygous mothers (where all four ordered genotypes of offspring come from the same genotype of mother and hence maternal effects cannot result in the appearance of a parent-of-origin dependent effect). A parent-of-origin dependent effect was attributed to a maternal effect if the sex by *i *effect depended significantly on the type of mother (i.e., there was a significant three-way interaction between sex, *i *and the type of mother, classed as heterozygote or homozygote).

### Significance testing of QTL

Our significance testing approach needed to take account of the autocorrelation of siblings within the F_3 _families. Therefore, we first calculated chromosome-wise and genome-wise threshold LOD scores for the sex-by-imprinting interaction in separate permutation procedures [48] for each of the traits ensuring that the specific family structure in this generation was maintained. For each trait, we achieved this by first calculating deviations of each individual from its family mean and randomly permuting these deviations within each family. We then randomly permuted all F_3 _family means and reconstructed new values for each individual by adding its permuted deviation to its new mean. Using these new values for each individual, we ran a canonical correlation analysis and computed the highest LPR score on each chromosome. This procedure was repeated 1000 times, and 5% chromosome-wise threshold values were obtained from the 50^th ^highest values generated for each chromosome. The 5% genome-wise threshold value for each trait was obtained from the 50^th ^highest value among the 1000 highest LPR values across all 19 chromosomes in each permutation run [48]. First simulations revealed that the genome- and chromosome-wise thresholds were very similar to those obtained using the effective number of markers method based on the eigenvalues of the marker correlation matrix [49]. Since the latter method allows for direct computation of the thresholds for all traits, whereas the simulation required significant computing time for each trait, we used the thresholds obtained using the effective number of markers. This method calculates the number of independent tests in a genome or chromosome scan and uses the "effective number of markers" in a Bonferonni correction. We used both the conservative genome-wise significance threshold as well as the chromosome-wise thresholds because this approach has been shown to give overall the best results by increasing the discovery of true positives while at the same time avoiding problems using the false discovery rate in gene mapping experiments [50].

After loci were identified using the genome- or chromosome-wide significance thresholds for the interaction model we used post-hoc tests to characterize the phenotypic patterns caused by genomic imprinting. We included all significant effects of QTL using a protected test where pleiotropic effects of QTL are included whenever the effect of a locus on other traits is significant at the pointwise (*p *< 0.05; LPR > 1.3) level. Thus, while we apply the stringent genome- or chromosome-wide threshold for QTL detection to minimize type I errors, we characterize the distribution of pleiotropic effects across the entire set of traits to minimize type II errors. The imprinting patterns were determined using the mixed model approach and are given by the relationship between the additive (*a*), dominance (*d*) and parent-of-origin genotype value (*i*) [36]. We distinguish a total of three different imprinting patterns: parental (paternal or maternal) expression, bipolar dominance and polar dominance, see above [36].

## Authors' contributions

JBW and JMC conceived experiment and data analysis. RH analysed data. JBW and LJL conducted significance testing and provided programs used for analyses. RH, JMC, LJM and JBW wrote the paper. All authors contributed to previous drafts of the paper and have approved the final manuscript.

## Supplementary Material

Additional file 1 **Sex-dependent imprinting effect QTL (*****Sbi*****QTL) and their patterns for body weights, growth and organ weights.** Coordinates (Mb) are based on mouse genome build 36 http://www.ensembl.org and location refers to F_2 _cM map distances. Patterns are listed as Paternal = paternal expression, Maternal = maternal expression, Bipolar = bipolar imprinting, Under = polar underdominance, Over = polar overdominance, NS = non-significant. Loci were identified when the LPR for the overall effect of a locus exceeded the chromosome-wide or genome-wide threshold and they showed a significant sex by *i *interaction effect. The genome-wide significance threshold is given at an LPR of 3.41 and the chromosome-wide thresholds are given for each locus (in italics) as is the highest LPR for the locus. Traits shown in bold indicate a QTL effect. Click here for file

Additional file 2 **The table lists the identified sex by imprinting QTL (*Sbi*QTL) and effects for males and females separately.** First, the *Sbi*QTL is identified, followed by the trait affected and by marker name, the F2 map location in cM and the genome coordinate based on mouse build 36 http://www.ensembl.org along with the confidence intervals. This is followed by the locus LPR and the chromosome-wide threshold (the genome-wide is 3.41), the estimates for the sex by *i *interaction, the corresponding standard error, the interaction LPR and the *r*^2 ^for the interaction effect. The next section details for males the additive *a*, dominance *d*, and imprinting *i *effects with their estimates, the estimates divided by the standard deviation and standard error as well as the corresponding LPR. Next, the imprinting patterns are given where 'Paternal' refers to paternal expression, 'Maternal' to maternal expression, 'Bipolar' to bipolar dominance, 'Over' to polar overdominance, 'Under' to polar underdominance and 'NS' to non-significant. This is followed by the genotypic values of the four ordered genotypes with their standard errors SE. We next present in the same order the equivalent information for females. Click here for file
