# Supplementary material for: Sex dependent imprinting effects on complex traits in mice
Source: BMC Evol Biol. 2008 Oct 31;8:303. doi: 10.1186/1471-2148-8-303 (PMC2605465; doi:10.1186/1471-2148-8-303)
Supplement: Additional file 1 — Sex-dependent imprinting effect QTL (SbiQTL) and their patterns for body weights, growth and organ weights. Coordinates (Mb) are based on mouse genome build 36 http://www.ensembl.org and location refers to F2 cM map distances. Patterns are listed as Paternal = paternal expression, Maternal = maternal expression, Bipolar = bipolar imprinting, Under = polar underdominance, Over = polar overdominance, NS = non-significant. Loci were identified when the LPR for the overall effect of a locus exceeded the chromosome-wide or genome-wide threshold and they showed a significant sex by i interaction effect. The genome-wide significance threshold is given at an LPR of 3.41 and the chromosome-wide thresholds are given for each locus (in italics) as is the highest LPR for the locus. Traits shown in bold indicate a QTL effect. [file 1471-2148-8-303-S1.doc]

**Table** 1: Sex dependent imprinting effect QTL (*Sbi*QTL) and their patterns for body weights, growth and organ weights.

| ***Sbi*QTL** | **Location** | **Coordinate** | **Trait** | **LPR** | **Pattern males** | **Pattern females** | *r^2^* [%] |
| --- | --- | --- | --- | --- | --- | --- | --- |
| ***Sbi1.1*** | 44.27 | 105.92 | **Tail** | 9.29  (*2.31*) | NS | Paternal | 1.22 |
| ***Sbi2.1*** | 47.72 | 82.25 | Spleen | 2.72  (*2.29*) | Over | NS | 1.64 |
| ***Sbi3.1*** | 74.45 | 13.30 | Week 3  Week 4  Week 5  Grow13 | 2.76  (*2.24*) | Bipolar  Bipolar  Bipolar  Bipolar | NS  NS  NS  NS | 0.63  1.23  0.91  0.74 |
| ***Sbi5.1*** | 59.72 | 124.68 | **Week 5**  Kidney  Heart | 4.16  (*2.19*) | Paternal  NS  NS | NS  NS  NS | 0.58  1.11  1.07 |
| ***Sbi6.1*** | 1.94 | 10.76 | **Week 4**  **Week 5**  Week 6  Week 7  Week 8  Week 9  Week 10 | 7.04  (*2.19*) | NS  NS  NS  NS  NS  NS  NS | Paternal  Paternal  Paternal  Paternal  Paternal  Paternal  Paternal | 3.28  2.90  1.53  1.51  1.28  1.01  0.75 |
| ***Sbi6.2*** | 41.96 | 94.76 | **Week 4**  **Week 5**  **Week 6**  **Week 7** | 13.28  (*2.19*) | NS  NS  NS  NS | NS  NS  Paternal  Paternal | 1.28  1.33  0.91  0.98 |
| ***Sbi7.1*** | 27.70 | 56.32 | **Week 4**  **Week 5** | 11.25  (*2.13*) | Paternal  Paternal | NS  NS | 0.71  0.62 |
| ***Sbi8.1*** | 7.35 | 18.26 | **Week 9**  Grow310  Liver | 4.88  (*2.13*) | NS  NS  NS | NS  NS  Over | 0.71  0.84  1.43 |
| ***Sbi8.2*** | 48.85 | 94.64 | Spleen | 3.26  (*2.13*) | Maternal | NS | 1.64 |
| ***Sbi9.1*** | 8.94 | 24.62 | Fatpad | 2.49  (*2.19*) | Paternal | NS | 2.57 |
| ***Sbi15.1*** | 25.96 | 62.91 | Week 2  Week 4  Week 5  Week 6  Week 7  Week 8  Week 9  Week 10  Grow310  **Fatpad**  Kidney  Heart  Liver | 3.86  (*2.06*) | NS  NS  Paternal  NS  NS  NS  NS  NS  NS  NS  Bipolar  NS  NS | NS  NS  NS  Ns  Under  Under  NS  Maternal  NS  Bipolar  NS  NS  NS | 0.80  1.54  1.85  1.88  1.93  2.67  2.36  2.34  1.95  3.42  1.83  1.07  1.29 |
| ***Sbi17.1*** | 36.94 | 67.48 | Week 2  Week 4  Week 5  Week 6  Week 7  Week 8  Week 9  Week 10  Grow310  Tail  Kidney | 2.44  (*2.06*) | NS  NS  NS  NS  NS  NS  NS  NS  NS  NS  NS | NS  NS  NS  NS  NS  NS  NS  NS  NS  NS  NS | 0.72  0.93  0.78  1.29  1.29  0.87  1.27  1.09  0.94  0.78  1.44 |
| ***Sbi19.1*** | 26.97 | 28.19 | **Spleen**  Fatpad  **Tail**  Heart | 6.94  (*1.99*) | NS  NS  NS  Bipolar | NS  NS  NS  NS | 1.05  1.05  0.91  1.42 |

Table 1 legend: Coordinates (Mb) are based on mouse genome build 36 (www.ensembl.org) and location refers to F_2_ cM map distances. Patterns are listed as Paternal = paternal expression, Maternal = maternal expression, Bipolar = bipolar imprinting, Under = polar underdominance, Over = polar overdominance, NS = non-significant. Loci were identified when the LPR for the overall effect of a locus exceeded the chromosome-wide or genome-wide threshold and they showed a significant sex by *i* interaction effect. The genome-wide significance threshold is an LPR of 3.41 and the chromosome-wide thresholds are given for each locus (in italics) as is the highest LPR for the locus. Values in the *r^2^* column indicate the proportion of phenotypic variance explained by the sex by imprinting interaction. Traits shown in bold indicate a QTL effect significant at the genome-wide level.
